# A novel blockchain-watermarking mechanism utilizing interplanetary file system and fast walsh hadamard transform

**DOI:** 10.1016/j.isci.2024.110821

**Published:** 2024-08-28

**Authors:** Tong Liu, Si-Nga Lai, Xiaochen Yuan, Yue Liu, Chan-Tong Lam

**Affiliations:** 1 Faculty of Applied Sciences, Macao Polytechnic University, Macao SAR 999078, China

**Keywords:** Natural sciences, Applied sciences, Computer science

## Abstract

This article proposes a new digital watermarking mechanism based on the Ethereum blockchain, Smart Contract, and Interplanetary File System (IPFS), with an enhanced Fast Walsh Hadamard Transform (FWHT) algorithm for watermark embedding and extraction. The proposed scheme aims to address the limitations of existing digital watermarking techniques, such as dependence on third-party platforms, by leveraging the decentralization feature of blockchain. The Smart Contract is used to manage the transaction between the parties involved in the watermarking process, while IPFS is used to store the watermark data. The enhanced FWHT algorithm is used to embed the watermark into the host image without affecting its visual quality. The results show that the proposed scheme outperforms the state-of-the-art algorithms in terms of both imperceptibility and robustness. Additionally, it demonstrates that our scheme can effectively resist various attacks. Therefore, our scheme can be a promising solution for image copyright protection, authentication applications, and image trading.

## Highlights

• Propose a blockchain watermarking solution for image copyright protection
• Optimize WHT to embed watermark information and improve image quality
• Images are encrypted before IPFS upload, ensuring protection against theft
• The system offers high security and decentralization, avoiding third-party reliance

## Graphical abstract

**Figure:**
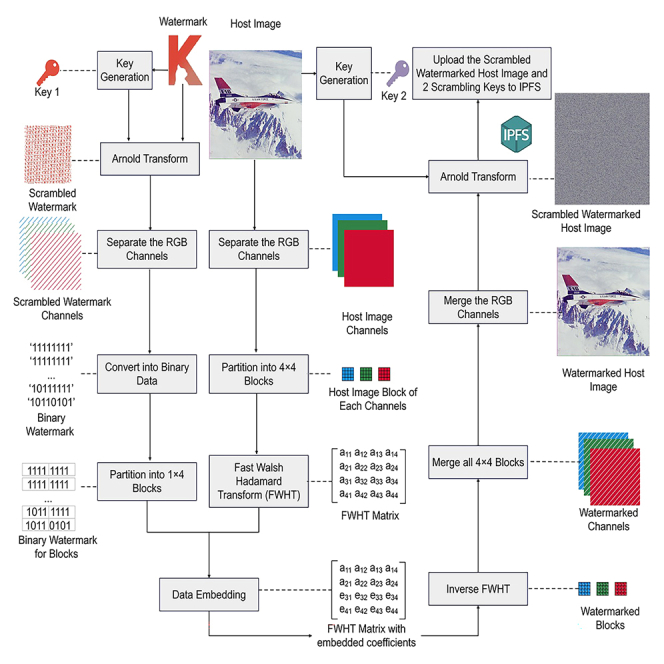

## Introduction

People will share their own digital artworks on today’s Internet, such as pictures, paintings, music, video, and so forth. That can help their artworks spread rapidly and widely, but in the meanwhile, it can also lead to massive misappropriation issues on the contrary. Because anyone may readily download, take a screenshot, or directly copy the relevant artwork, preventing misappropriation poses a significant challenge. In order to solve the problem and protect image copyright, some people will print the copyright information on the protected images directly as a watermark. On the one hand, the visible watermark may affect the image quality. On the other hand, the copyright information may cause privacy leakage. In order to solve these problems, digital watermarking technology was developed to safeguard intellectual property rights and prevent the problem of misappropriation using an invisible watermark.^1^^,^^2^^,^^3^ This refers to embedding particular information into digital signals, which can be either speeches, images, or videos. When the watermarked signal is copied, the embedded information is transferred along with it. However, a new consideration has arisen when using digital watermarking – that is, attaching digital watermarks to creative works necessitates the use of a trustworthy third-party platform. This platform is intended to establish ownership of artworks and to prevent illegal abuse, resale, and misappropriation.^4^^,^^5^^,^^6^ The entire procedure of digital watermark certification and validation must remain as safe and available. If this third-party platform goes offline, whether it is under maintenance or closed, the service will be unavailable or even cause data lost, leading to the failure of certification or validation of the digital watermark.

The new blockchain technology is suited for addressing the issue of long-term preservation and security authentication without the need for a third-party platform.^7^^,^^8^^,^^9^^,^^10^ This is because blockchain technology can store and share information in a transparent and decentralized way, which is supported by technologies such as cryptography and consensus processes.^11^^,^^12^^,^^13^^,^^14^ A peer-to-peer network that stores enormous volumes of transaction data using chains of blocks. Each block contains the encrypted hash of the previous block, as well as the creation date and transaction data. Because of the distinctive architecture, it is difficult to tamper with the block content. The transactions between two traders are recorded on the blockchain-connected distributed ledger, and these transactions can be permanently stored and validated. Although copyright information can be stored in the blockchain directly, due to the transparent nature of blockchain, everyone can access this information easily, leading to a risk of privacy leakage.^15^^,^^16^^,^^17^^,^^18^ Watermarking technology can provide an additional layer of privacy protection by hiding the copyright information within the cover image. The main objective of our work is to propose a mechanism that protects image copyright information without compromising privacy, and at the same time avoids the reliance of third-party platforms. The combination of watermarking and blockchain technology perfectly satisfies our requirements.

Based on the idea of combining blockchain technology with digital image watermarking, there exists a solution that uses Ethereum blockchain with smart contracts, Interplanetary File System (IPFS), and Zero watermarking algorithm for securing image storage, trading, and authentication.^19^^,^^20^^,^^21^ This framework achieves the concept of decentralization that is not required to rely on third-party authority for arbitration, and which has higher system availability and better cost effectiveness compared to the traditional watermarking approach. However, due to the characteristic of zero watermarking, the capability to protect the original host image against misappropriation has been lost. Therefore, in order to prevent misappropriation during image trading, we propose a watermarking method that practically embeds the ownership information into the host image while keeping it imperceptible. The enhanced framework can be explored as follows.

(1) An enhanced solution for blockchain watermarking is proposed, which uses a blind color watermarking algorithm with Fast Walsh Hadamard Transform (FWHT). The watermarked image will be protected by additional scrambling with a secret key before uploading to IPFS. The proposed solution provides strong protection against data tampering and data leakage for image trading. Additionally, it also has the ability to resist image misappropriation at the same time.
(2) An improved data embedding approach is proposed to enhance the original Walsh Hadamard Transform (WHT) algorithm proposed by Prabha et al..^22^ The new embedding approach is optimized to reduce the changes in the original host image while keeping the same effort, thus generating a watermarked image with better performance in terms of image quality and robustness.

The rest article is organized as follows: firstly related works introduces some state-of-the-art works combining watermarking and blockchain techniques. Next, the specific methodology of our proposed scheme is detailed in proposed method. Then the performance of the proposed solution in terms of image robustness, image quality and imperceptibility, and cost consumption is presented and analyzed in results and discussion. Finally, in conclusions and future work we make a conclusion and discuss the potential future works.

## Related works

In past years, traditional watermarking technology has been widely used in protecting intellectual property rights. The information could be encrypted using this technology and embedded into the image, which has a great impact on data privacy protection.^23^ The watermark taxonomy can be classified into robust and fragile.^24^^,^^25^^,^^26^ The robust watermark has good robustness against kinds of attacks, which can be used for copyright protection. The fragile watermark is sensitive to any tiny changes and is commonly used for integrity verification.^27^^,^^28^ In 2016, Liu et al.^29^ proposed a dual watermarking scheme for protecting the copyrights of color images by combining robust and fragile watermarking. This scheme is able to achieve image authentication and copyright protection at the same time. The robust watermark is embedded into the YCbCr color space, while the fragile watermark is embedded into the RGB spatial domain. The experimental results reveal that this method is able to resist attacks and can accurately detect the tampered area within the images. However, the dual embedding mechanism will affect the imperceptibility of watermarks. When the parameter *k* is set as 0.4, the average Peak signal-to-noise ratio (PSNR) of watermarked images is only 31dB. To improve the imperceptibility, in 2017, Makbol et al.^30^ proposed a new robust watermarking scheme based on Singular Value Decomposition (SVD). Different from the other SVD-based watermarking schemes, this method used singular vectors and singular values as secret keys, which is useful in avoiding the false positive problem. In addition, a Multi-Objective Ant Colony Optimization (MOACO) method was used here to improve the robustness of watermarking. The experimental results demonstrate that this scheme has excellent robustness and imperceptibility. In 2023, Sun et al.^31^ proposed a reversible robust watermarking scheme based on zernike moments and histogram shifting. The scheme chose two regions in the images for watermarking embedding, thus both the robustness and imperceptibility of the watermark can be guaranteed. This method is good at resisting geometric deformations. However, the precision of feature point extraction could be further improved. In 2024, Nie et al.^32^ proposed a compression-resistant model watermarking scheme called CRMW for model copyright protection. This scheme generated a robust watermark dataset utilizing image watermarking and compression algorithms for training. A back-door trigger was concealed in this framework, which made it robust to resist copyright theft. The experimental results indicate that this work has good robustness against the attacks of fine-pruning, neural cleanse, and STRIP. In 2024, to protect the security of images, Wen et al.^1^ proposed an encryption algorithm. They embedded the feature value and ciphertext into the cover images, so the receivers could decrypt ciphertext without additional secret keys. This method shows its effectiveness in improving the security of images, but can not verify the copyright of images.

Although the above technologies have their superiorities, their applications still need third-party platforms, which play an important role in content distribution, as well as data management and monitoring. In order to reduce the reliance on third-party platforms, several blockchain-based digital watermarking systems have been proposed. The blockchain technique can store and share information in a transparent and decentralized way, enhancing the reliability of watermarks without requiring reliance on a single third-party platform for storage and monitoring. In 2019, Liu et al.^33^ proposed an optimized image watermarking method based on Hessenberg Decomposition (HD) and SVD in the Discrete Wave Transformation (DWT) domain. This algorithm hides the watermark data under multiple transformation and decomposition techniques, resulting in high invisibility for the embedded watermark, and strong robustness for the watermarked image against various image attacks. However, such proposed method supports only the greyscale host image and watermark image; another drawback of the method is that the watermark extraction process requires parts of the information to be outputted from the embedding process, such as the embedded singular matrix of the host image and the singular matrices of the original watermark image, from the SVD algorithm. Additional resources and efforts are required for storing, transmitting, and securing such information when considering the ownership validation on an image trading platform. To overcome this problem, in 2022, Prabha et al.^22^ proposed an effective robust, and imperceptible color image watermarking method. It is a blind watermarking algorithm that does not require extra information to be provided during the watermark extraction, and it also supports the processing of color images. The algorithm embeds the watermark image to the host image by applying WHT, which is an orthogonal function consisting of values plus one or minus one only. Following its procedure, the algorithm subdivides the host image into 4×4 non-overlapping blocks, which are then transformed using WHT. The binary data converted from the scrambled watermark image is split up and embedded in the third and fourth rows of each sub-block or the host image using the proposed technique of WHT coefficients calculation, as a slight change in those rows may not profoundly affect the visual quality of an image. Finally, an inverse WHT will be applied to the processed host image and then combine all sub-blocks to obtain the watermarked host image. In 2020, Wang et al.^19^ proposed a secure image protection system using zero watermarks implemented in blockchain. This system utilizes IPFS, Ethereum, and image zero-watermark technologies to provide a platform for image trading securely. The platform uses Ethereum to access vital information and uses IPFS and the deployed smart contracts on the Ethereum blockchain to make images lossless and more secure. The Zero-watermark algorithm used in this framework will generate a zero-watermark image for later validation based on the host image, watermark image, and secret keys. The advantage of such an approach is that the watermark will not be embedded in the original host image and therefore, it is lossless on the image quality. However, regarding a platform for image trading, when a user retrieves an image without embedding any information about the original image owner, the problems of misappropriation might be derived from the later uses. It is instead requiring a watermarking algorithm that embeds a watermark image (as the owner information) to the host image but still needs to be imperceptible, as well as robustness for the watermarked image. In 2023, kallapu et al.^34^ proposed a searchable encryption system based on blockchain and attribute, which was capable of protecting data privacy. The contribution of this work is that the system combines attribute-based encryption, searchable encryption, and blockchain technology to offer fine-grained access control.

## Proposed method

This article proposes a novel Blockchain-Watermarking Mechanism (BWM) utilizing IPFS and FWHT. The watermarking method embeds the watermark data to the host image by applying the FWHT with the selected coefficients on the third and fourth rows of each image block, resulting in only slight visual variation to the original image, as well as getting strong robustness of the image against attacks. By utilizing the features of blockchain, Smart Contract, and IPFS, the watermarked image will be securely stored in a decentralized way and transmitted in a secure manner under reliable access control. In this way, the watermarking validation will be trustable without relying on any third-party authority. In this section, the system design, system workflows, and the algorithm of watermark embedding and extraction using FWHT are explained in detail.

### System design

**Figure 1:**
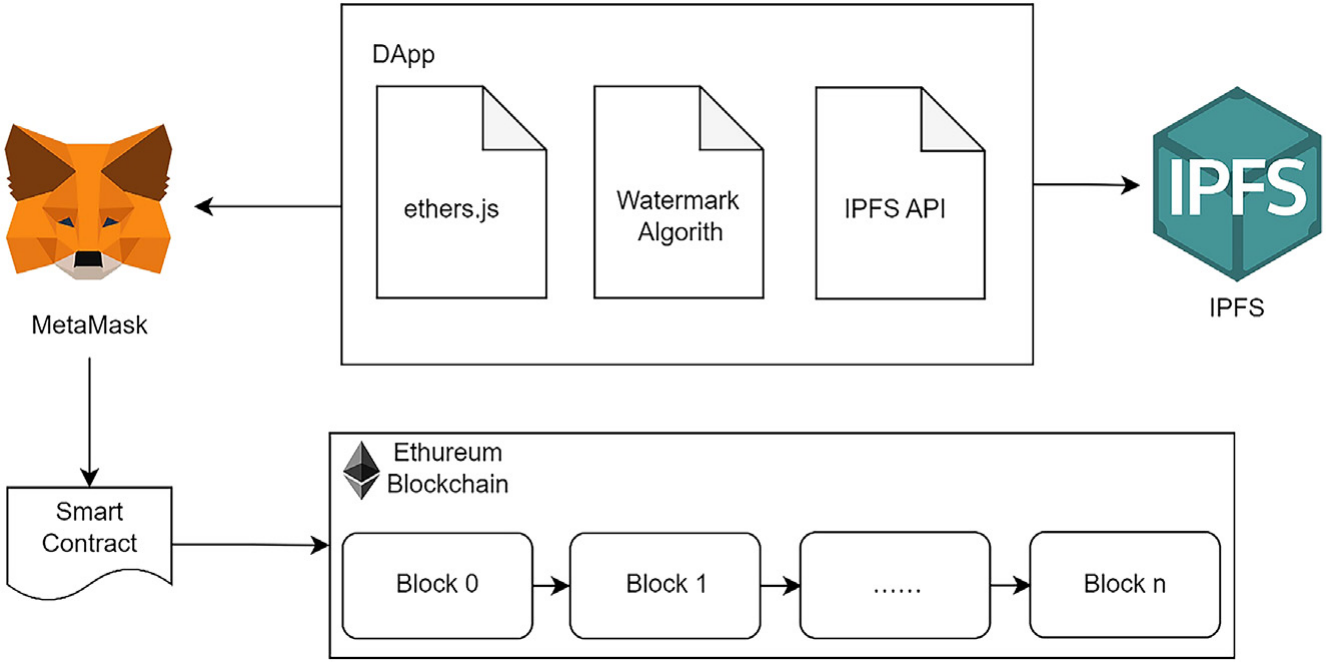
Architecture of the proposed Blockchain-Watermarking Mechanism (BWM)

The proposed method is implemented with a Decentralized App (DApp). There are two subjects involved in the work trading process – image owner (Owner) and image requester (Requester). The DApp makes use of a Smart Contract as the intermedia between Owner and Requester for image trading requests, authorization, deposit, and transmission for the images and the secret keys used for data encryption and image scrambling. IPFS plays the role of storage to keep the images and keys transmitting between the Owner and Requester, where it is connected by IPFS API from the DApp, and all the transactions are recorded in the blockchain transaction blocks on Ethereum. The owner and Requester will interact with the Smart Contract via the crypto wallet MetaMask from the client side using the library ethers.js and make use of its asymmetric cryptography functions and keys for data encryption and decryption. The DApp is also to handle the watermark embedding and extraction for the trading images using the FWHT algorithm. Figure 1 illustrates the system architecture.

The entire system consists of two process workflows. The first process workflow is the launch flow for the Owner, where the Smart Contract is deployed prior to the works trading process. The second process workflow is the request flow about the Requester requesting, obtaining, and validating the ownership of the watermarked host image *WI*. It also involves Owner authorizing the Requester, embedding the watermark to the host image, and transmitting it to the Requester.

**Figure 2:**
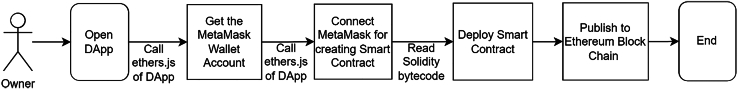
Launch flow in the proposed BWM

In the launch flow, the Owner will access the DApp and connect to the MetaMask wallet account by using the ethers.js library integrated with it. The owner will initiate a Smart Contract deployment request from the DApp via the MetaMask account. The Smart Contract contains the owner information, requirement of deposit, and authorization list which is initially empty, and provides the search function for the authorized Requester to obtain the access information for the watermarked image. After the deployment request is done mining from the Ethereum network, it will be deployed to the Ethereum blockchain, and the contract address will be returned. Figure 2 illustrates the launch flow for Smart Contract deployment.

In the request flow, the Requester can view the degraded host image and request to retrieve it via Ethereum blockchain and IPFS. Requester will not get access until the Owner grants approval and adds the Requester to the authorization list in the Smart Contract. Requester will connect to the MetaMask wallet account in the DApp to generate their own public key and complete the deposit with the public key via the Smart Contract. Once the Owner confirms the deposit completed by the Requester via the function defined in the Smart Contract, the Owner will use the host image and a watermark image to generate the scrambling keys SK and the scrambled watermarked host image SWI using the FWHT watermark embedding algorithm via the DApp. The Owner uses the public key provided by the Requester to encrypt SK, and then uploads these encrypted keys ESK and SWI to the IPFS. The Owner also records the IPFS location address for further authorization. Finally, the Owner will add the Requester’s account together with the IPFS address into the authorization list in the Smart Contract, as well as share this information with the Requester by sending a transaction hash via the Ethereum blockchain.

**Figure 3:**
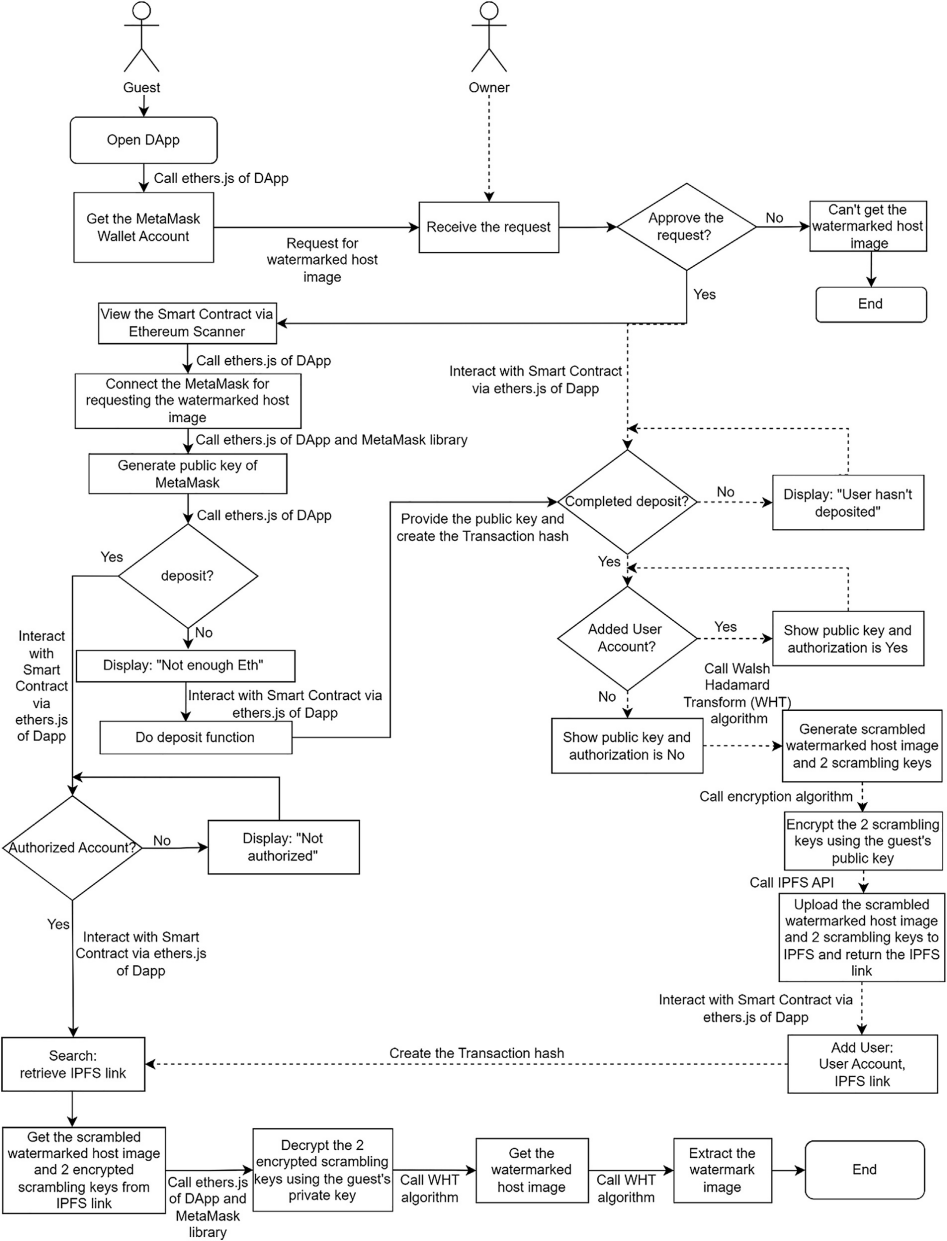
Requester flow in the proposed BWM

Once the Requester receives the transaction hash, the Requester is able to get the ESK and the IPFS address of SWI from the Ethereum blockchain. The requester can obtain the original SK by connecting to the MetaMask to decrypt the ESK using their own private key. Up to this stage, Request is now able to recover the WI from SWI through the unscrambling function in the DApp by providing SK. Furthermore, Requester is also allowed to validate the ownership of WI in the DApp using the FWHT watermark extraction algorithm in the DApp. Figure 3 illustrates the entire process of the requester flow.

### Smart contract for image trading

The proposed scheme used a smart contract that is simply a program stored on the Ethereum blockchain. It acts as an intermediary to complete the actions by following the instructions defined in the contract. The contract contains owner information, an authorization list, a user info list, deposit function, and a search function. The purpose of the deposit function is to receive Ether sent by the user, store the public key provided by the user, mark the user as authorized, and update the user’s Ether balance. By calling this function, users can deposit Ether into the contract while providing their public key for contract records and verification. The search function is designed to retrieve the IPFS address stored in the smart contract. It returns a structure containing a string field, representing the IPFS address stored in the smart contract. Those functions are predefined in the binary codes and stored in the DApp. The owner will deploy the smart contract to Ethereum based on these binary codes. The deposit function is the first step for the Requester to commit their request to get the right of image usage. It enables users to interact with the contract by depositing Ether and providing their public key for further operations within the contract. It is marked as payable, meaning it can receive Ether transfers. When someone calls this function, their public key will be saved in the contract. The predefined price in Ether is required to complete the deposit, at the same time the Requester will be asked for permission to generate their public key from the MetaMask wallet, to send out together with the deposit transaction. After the mining is done by the Ethereum network, the user address and the public key to the Requester will be stored in the user info list in the Smart Contract.

The user info list contains the information for those users who have already deposited and provided their public key for later data encryption. The owner will base on this list to validate whether the requester completed the deposit or not, to continue the next step of adding the Requester to the authorization list. The authorization list in the Smart Contract allows the Owner to add or remove the Requester’s account address to control who can access the contract and use the search function to obtain the result. The list will store the user address, and the IPFS address that contains the watermarked image in scrambled form, and those two ESK. If the Requester has not completed the deposit with the public key provided through the deposit function, the search function will not return the information of the IPFS address. Only after the Requester completes the deposit and is added to the authorization list by the Owner, the search function called by the Requester will return the correct information of the IPFS address.

### Image embedding using fast walsh hadamard transform

**Figure 4:**
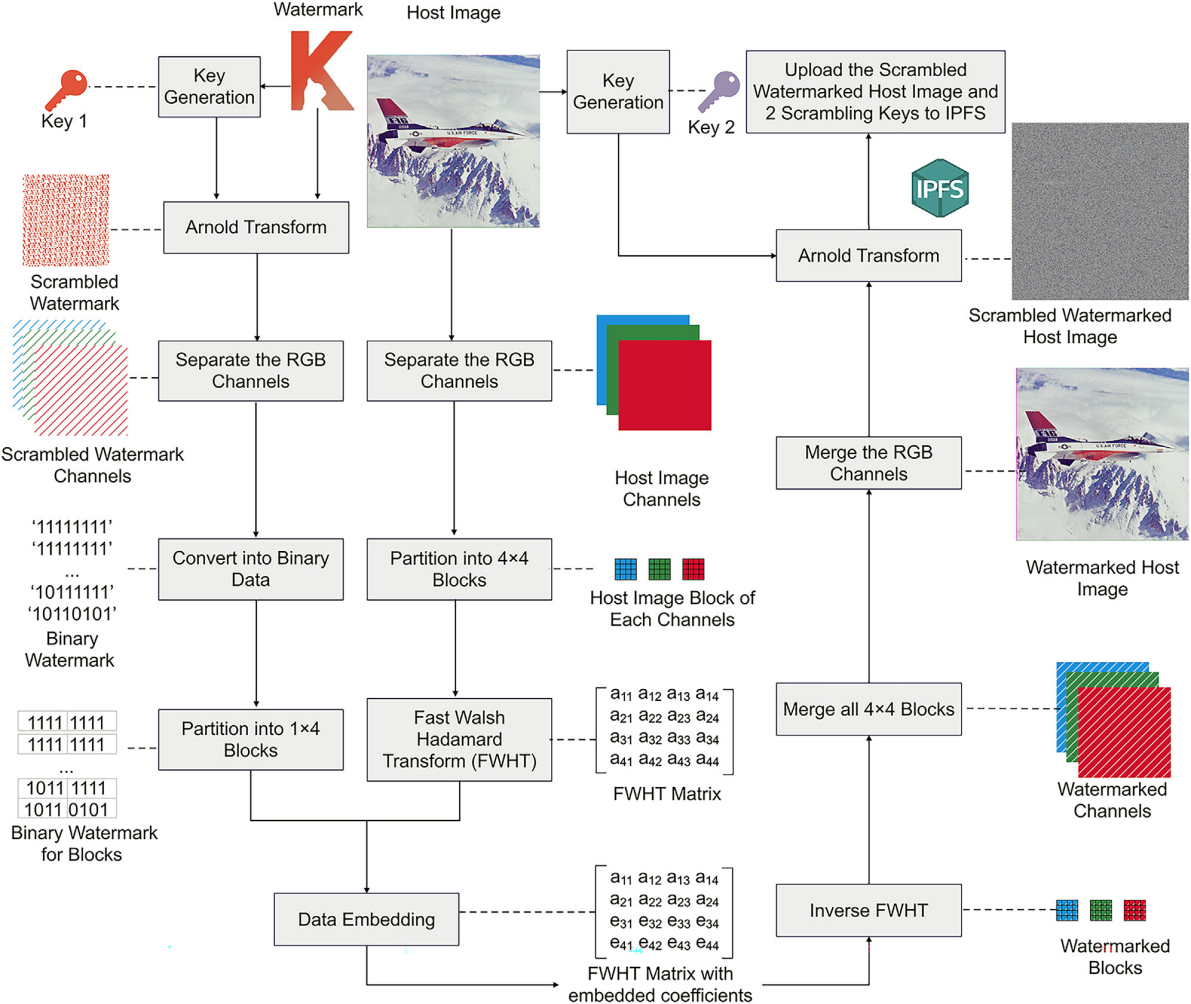
Process of watermark embedding using fast Walsh Hadamard transform

After the Requester completes the deposit and provides the public key, the Owner will embed a watermark image, which represents the ownership information in binary form, into the host image using the DApp. Firstly, the host image is split into its RGB channels, and then the numerical values of each channel are transformed from decimal to binary form. Next, the host image is divided into blocks of 4×4 on each channel respectively, and then the FWHT algorithm is applied to each block. To make the watermarked image more imperceptible, the third and fourth rows of the FWHT matrix are selected for information embedding. This is because the slight change in the elements of these two rows will not significantly degrade the image quality. After determining the embedding positions, the embedding data are calculated based on the parity of the differences calculated by two embedding FWHT rows and scrambled binary values in the watermark image *W*. To generate the scrambled binary value in *W*, the Arnold Transform is firstly applied to *W* with randomly generated Key1, and then the value of the resulting scrambled watermark in each channel is converted from decimal to binary form. Sequentially, the information is embedded by changing the coefficients in the corresponding positions of the FWHT matrix with generated embedding data. Finally, to generate the watermarked host image WI, the inverse FWHT is employed for each block first, and all the blocks and channels are then merged together. To protect the resulting image from unauthorized access, the WI is scrambled by the Arnold Transform with Key2, thus resulting in the scrambled watermarked host image SWI. Only the person who has those keys can recover WI from SWI or extract *W* from WI. The process is shown in Figure 4, and the detailed steps are introduced in Algorithm 1 as follows:

Algorithm 1 **Watermark embedding using FWHT**

 **Input:** Host Image *I*, Watermark Image *W*

 **Output:** Scrambled Watermarked Host Image SWI, Key1, Key2

 **Step 1:** Apply Arnold Transform to scramble *W* with a randomly generated key Key1 to obtain the Scrambled Watermark Image SW.

 **Step 2:** Split *W* into R, G, and B channels to process separately. Convert each pixel of every channel of SW into binary data SWB.

 **Step 3:** Partition SWB into 1×4 blocks containing 4 bits in each.

 **Step 4:** Split *I* into R, G, and B channels and partition them into non-overlapping blocks with each having 4×4 pixels.

 **Step 5:** Apply the FWHT for each block of *I*.

 **Step 6:** Embed each set of binary data from SWB into the FWHT coefficients of each block from *I* to get the FWHT embedded coefficients for all subblocks.

 **Step 7:** Apply the inverse FWHT for each FWHT matrix of 4×4 image block with embedded FWHT coefficients and take the floor values.

 **Step 8:** Merge all the 4×4 subblocks obtained in step 5 and merge the R, G, and B channels to get the WI.

 **Step 9:** Apply another Arnold Transform with a randomly generated key Key2 for WI to output SWI, together with Key1 and Key2.

In the step of binary data embedding to the FWHT matrix, considering an FWHT matrix of an image sub-block as,

(Equation 1) $$FWHT\left(U,V\right)=\left[\begin{array}{cccc} a_{11} & a_{12} & a_{13} & a_{14}\\ a_{21} & a_{22} & a_{23} & a_{24}\\ a_{31} & a_{32} & a_{33} & a_{34}\\ a_{41} & a_{42} & a_{43} & a_{44} \end{array}\right]$$

as the slight change on the third and fourth row elements of the 4×4 FWHT matrix of an image block will not have an excessively effect on the quality of the image, we will select those coefficient sets ($a_{31},a_{41}$), ($a_{32},a_{42}$), ($a_{33},a_{43}$) and ($a_{34},a_{44}$) to embed each 4-bit data extracted from the watermark image.

Firstly, the algorithm calculates the difference values of the FWHT coefficients for every 4×4 block in the third row and the fourth row, which is shown as follows:

(Equation 2) $$D_{i}=\lfloor \left|a_{3i}-a_{4i}\right|\rfloor$$

$a_{3i}$ are the coefficients in the third row, where $a_{3i}=a_{31},a_{32},a_{33},a_{34}$; $a_{4i}$ are the coefficients in the fourth row, where $a_{4i}=a_{41},a_{42},a_{43},a_{44}$; and where ⌊…⌋ is the floor function, |…| is the absolute function, $D_{i}$ the difference values. The data embedded coefficients are calculated by: If $D_{i}$ mod 2 = 0

(Equation 3) $$l_{i}=\left\{ \begin{array}{cc} \min\left(a_{3i},a_{4i}\right) & if\;b_{i}=0\\ \min\left(a_{3i},a_{4i}\right)-0.5 & if\;b_{i}=1 \end{array}\right.$$

(Equation 4) $$m_{i}=\left\{ \begin{array}{cc} \max\left(a_{3i},a_{4i}\right) & if\;b_{i}=0\\ \max\left(a_{3i},a_{4i}\right)+0.5 & if\;b_{i}=1 \end{array}\right.$$

If $D_{i}$ mod 2 = 1

(Equation 5) $$l_{i}=\left\{ \begin{array}{cc} \min\left(a_{3i},a_{4i}\right)-0.5 & if\;b_{i}=0\\ \min\left(a_{3i},a_{4i}\right) & if\;b_{i}=1 \end{array}\right.$$

(Equation 6) $$m_{i}=\left\{ \begin{array}{cc} \min\left(a_{3i},a_{4i}\right)+0.5 & if\;b_{i}=0\\ \min\left(a_{3i},a_{4i}\right) & if\;b_{i}=1 \end{array}\right.$$

where $b_{i}$ is the binary data to embed, $l_{i}$ and $m_{i}$ are the nominated data for embedded coefficients. Finally, the embedded coefficients are assigned using the values of $l_{i}$ and $m_{i}$ and arranged according to the values of $a_{3i}$ and $a_{4i}$ as:

(Equation 7) $$e_{3i}=\left\{ \begin{array}{cc} m_{i} & if\;a_{3i}\geq a_{4i}\\ l_{i} & if\;a_{3i}<a_{4i} \end{array}\right.$$

(Equation 8) $$e_{4i}=\left\{ \begin{array}{cc} m_{i} & if\;a_{4i}>a_{3i}\\ l_{i} & if\;a_{4i}\leq a_{3i} \end{array}\right.$$

$e_{3i}$ and $e_{4i}$ are the finalized embedded coefficients. Thus, the Fast Walsh Hadamard matrix of a 4×4 image sub-block with embedded coefficients is:

(Equation 9) $$eFWHT\left(U,V\right)=\left[\begin{array}{cccc} a_{11} & a_{12} & a_{13} & a_{14}\\ a_{21} & a_{22} & a_{23} & a_{24}\\ e_{31} & e_{32} & e_{33} & e_{34}\\ e_{41} & e_{42} & e_{43} & e_{44} \end{array}\right]$$

The data embedding in the algorithm makes use of the characteristics of the remainders while dividing odd and even numbers by two. It ensures the differences between the FWHT coefficients in the third and fourth rows match with what binary data is embedded to. Based on the defined algorithm, the data embedding for each FWHT coefficient will at most change the original value only with either +0.5 or −0.5 in all the cases.

### Watermark extraction using fast walsh hadamard transform

The image extraction in the proposed method will be performed by Requester, to obtain the original WI and to validate the ownership of WI. The requester will obtain the SWI, and the encrypted Key1 and Key2 from IPFS, and then utilize the private key to decrypt those two keys. With those decrypted keys on hand, WI can be obtained by applying inverse Arnold Transform to unscramble SWI using Key2 through the DApp. Similar to the embedding process, the WI is divided into 4×4 blocks in each channel and the coefficient matrix is then produced by applying FWHT on WI in the DApp. Next, the watermark information is extracted based on the parity of the differences, which are calculated between the third and fourth rows in the FWHT coefficient matrix.

**Figure 5:**
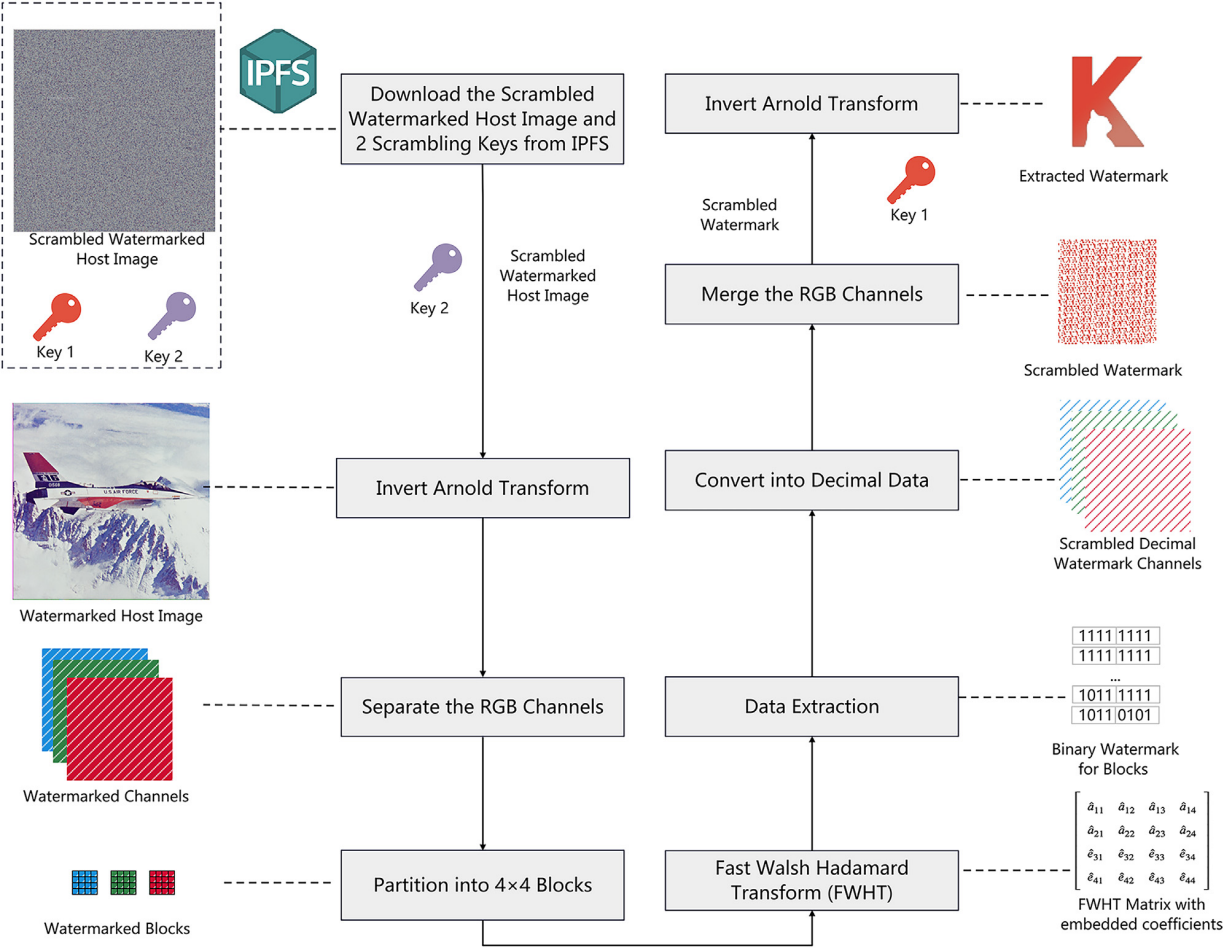
Process of watermark extraction using fast Walsh Hadamard transform

The scrambled watermark image SW can be produced by merging all the extracted data after converting it from binary to decimal form. Finally, the extracted watermark image EW is obtained by applying the inverse Arnold Transform to SW using Key1. The process is shown in Figure 5, and the detailed steps are shown in Algorithm 2.

Algorithm 2 **Watermark extraction using FWHT**

 **Input:** Scrambled Watermarked Host Image SWI, Key1, Key2

 **Output:** Extracted Watermark Image EW

 **Step 1:** Apply inverse Arnold Transform for SWI based on Key2 to recover the original WI.

 **Step 2:** Split WI into R, G, and B channels to process separately.

 **Step 3:** Partition each channel of WI into non-overlapping blocks with each having 4×4 pixels.

 **Step 4:** Apply the FWHT for each sub-block of WI.

 **Step 5:** Extract the data based on the difference values between FWHT coefficients in the third row and the fourth row. Calculate the remainder of the difference values by dividing two to get the binary data.

 **Step 6:** Merge all the extracted 1×4 binary data blocks and convert them into decimal data for each channel.

 **Step 7:** Merge the R, G, and B channels to reconstruct the SW.

 **Step 8:** Apply inverse Arnold Transform on SW with Key1 to get EW.

For an FWHT matrix of a 4×4 image sub-block:

(Equation 10) $$FWHT\left(U,V\right)=\left[\begin{array}{cccc} {\hat{a}}_{11} & {\hat{a}}_{12} & {\hat{a}}_{13} & {\hat{a}}_{14}\\ {\hat{a}}_{21} & {\hat{a}}_{22} & {\hat{a}}_{23} & {\hat{a}}_{24}\\ {\hat{e}}_{31} & {\hat{e}}_{32} & {\hat{e}}_{33} & {\hat{e}}_{34}\\ {\hat{e}}_{41} & {\hat{e}}_{42} & {\hat{e}}_{43} & {\hat{e}}_{44} \end{array}\right]$$

The algorithm calculates the difference values between ${\hat{e}}_{3i}$ and ${\hat{e}}_{4i}$

(Equation 11) $${\hat{D}}_{i}=\lfloor \left|{\hat{e}}_{3i}-{\hat{e}}_{4i}\right|\rfloor$$

where, ${\hat{e}}_{3i}$ and ${\hat{e}}_{4i}$ are the FWHT embedded coefficients in the third and fourth rows of the FWHT matrix, and ${\hat{D}}_{i}$ is the difference in values. The hidden data ${\hat{b}}_{i}$ are extracted from the values of ${\hat{D}}_{i}$ by:

(Equation 12) $${\hat{b}}_{i}=\lfloor {\hat{D}}_{i}\rfloor \mathrm{mod}\;2$$

Therefore, when the difference value between ${\hat{e}}_{3i}$ and ${\hat{e}}_{4i}$ is an odd number, the extracted binary will be a 1; otherwise, it will be a 0.

## Results and discussion

**Figure 6:**
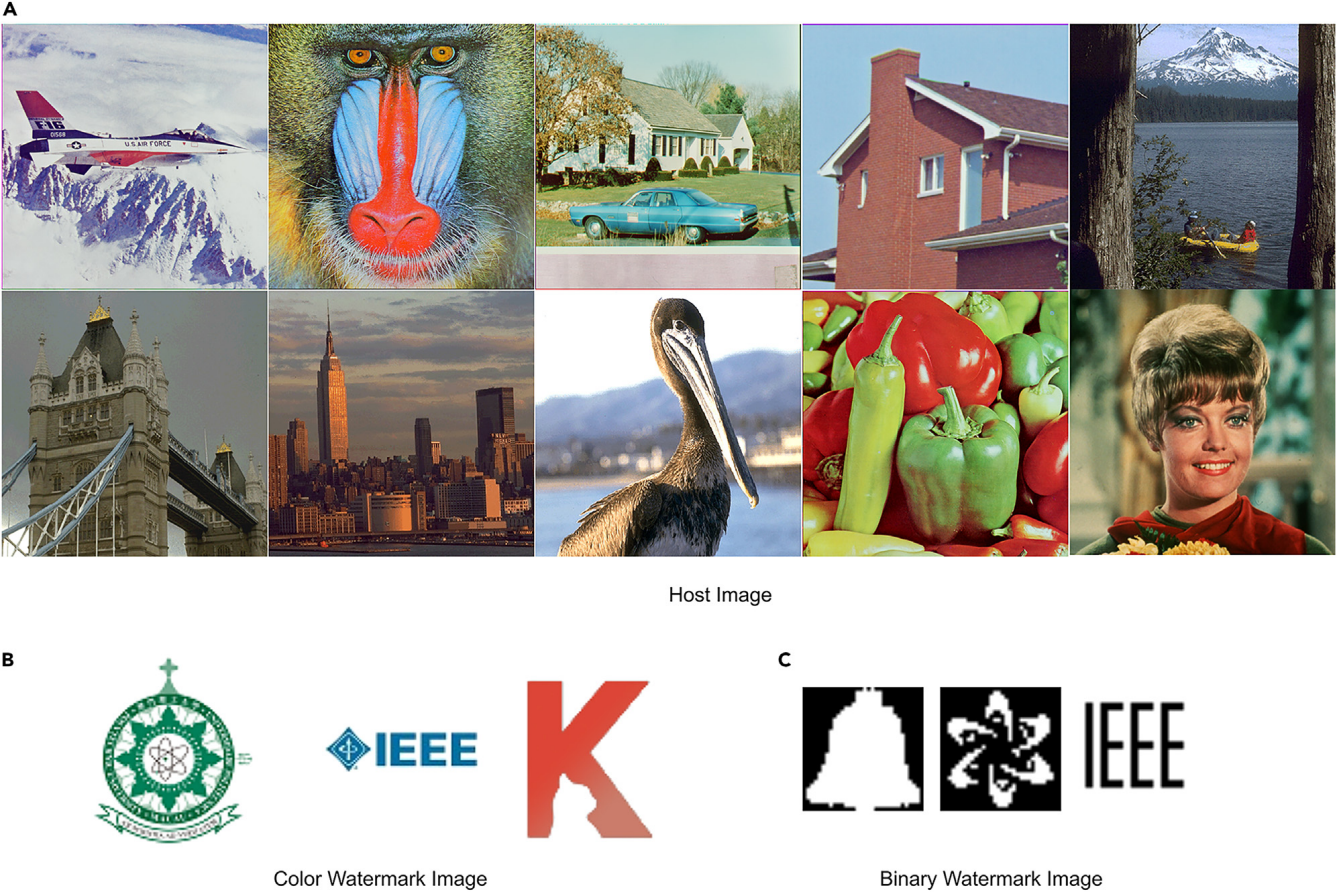
Examples of cover images and watermark images (A) Host images, (B) Color watermark images, and (C) Binary Watermark images.

In this section, a lot of experiments are conducted to test the performance of the proposed BWM in terms of image quality, robustness under attacks, embedding capacities, and the costs of using blockchain in the smart contract. All the experiments are implemented on a macOS environment with an Apple M2 CPU and 16 GB RAM, using Python 3.10.9 and MATLAB R2022b. We use USC - SIPI Image Database^35^: http://sipi.usc.edu/database/database.php and CVG - UGR Image Database^36^: https://ccia.ugr.es/cvg/dbimagenes/ to test the performance of our proposed scheme. Figure 6 shows some selected examples of cover images and watermark images: Figure 6A displays ten 24-bit color host images, which of size 512×512 “airplane,” “baboon,” “house,” “house2,” “manhatan,” “london,” “lostlake,” “pelican,” “peppers,” and “zelda”; Figure 6B is the three 24-bit color watermark images in the size of 90×90: “MPU,” “IEEE,” and “K2,” and Figure 6C is the three binary watermark images, which of size 32×32: “FDCT,” “IEEE,” and “Bell.”

In order to objectively evaluate the quality of images, we use three different metrics *PSNR*, *WPSNR*, and *SSIM*, which are defined in (13), (15), and (18) respectively. On the other hand, to evaluate the robustness of our scheme under different attacks, we use NC and BER, as defined in (19) and (20), respectively.

(Equation 13) $$PSNR=10\times \log_{10}\left(\frac{\max\;{\left(I\left(x,y,z\right)\right)}^{2}}{MSE}\right)$$

where MSE represents the mean square error between I(x,y,z) and W(x,y,z), which is:

(Equation 14) $$MSE=\frac{1}{3\times M\times N}\sum_{z=1}^{3}\sum_{x=1}^{M}\sum_{y=1}^{N}{\left[I\left(x,y,z\right)-W\left(x,y,z\right)\right]}^{2}$$

(Equation 15) $$WPSNR=10\times \log_{10}\left(\frac{\max\;{\left(I\left(x,y,z\right)\right)}^{2}}{WMSE}\right)$$

(Equation 16) $$WMSE=\frac{1}{3\times M\times N}\sum_{z=1}^{3}\sum_{x=1}^{M}\sum_{y=1}^{N}NVF\times {\left(I\left(x,y,z\right)-W\left(x,y,z\right)\right)}^{2}$$

where NVF is a normalized function which is calculated as (17),

(Equation 17) $$NVF=\frac{1}{1+\theta \sigma_{I\left(x,y,z\right)}^{2}}$$

(Equation 18) SSIM=L(W,I)×C(W,I)×S(W,I)

where L(W,I) is the luminance comparison, C(W,I) is the contrast comparison, and S(W,I) is a saturation comparison.

(Equation 19) $$NC=\frac{\sum_{z=1}^{3}\sum_{x=1}^{m}\sum_{y=1}^{n}W\left(x,y,z\right)\times \hat{W}\left(x,y,z\right)}{\sqrt{\sum_{z=1}^{3}\sum_{x=1}^{m}\sum_{y=1}^{n}W{\left(x,y,z\right)}^{2}}\times \sqrt{\sum_{z=1}^{3}\sum_{x=1}^{m}\sum_{y=1}^{n}\hat{W}{\left(x,y,z\right)}^{2}}}$$

(Equation 20) $$BER=\frac{1}{P\times Q}\left(\sum_{i=1}^{P}\sum_{j=1}^{Q}{\left[W\left(i,j\right)-W^{*}\left(i,j\right)\right]}^{2}\right)$$

As for the embedding capacity, here we use bit-per-pixel (bpp) to describe the mean value of number of bits stored in each pixel in the host image. For a host image in size P×Q and a watermark image in size M×N, bpp is calculated as follows:

(Equation 21) $$bpp=\frac{M\times N\times b}{P\times Q\times c}$$

where *c* is the number of channels of the host image and *b* is the number of bits of the watermark image. For a given watermark image, the payloads of the watermarked image decrease as the size of the cover image increases.

### DApp implementation and security analysis

**Figure 7:**
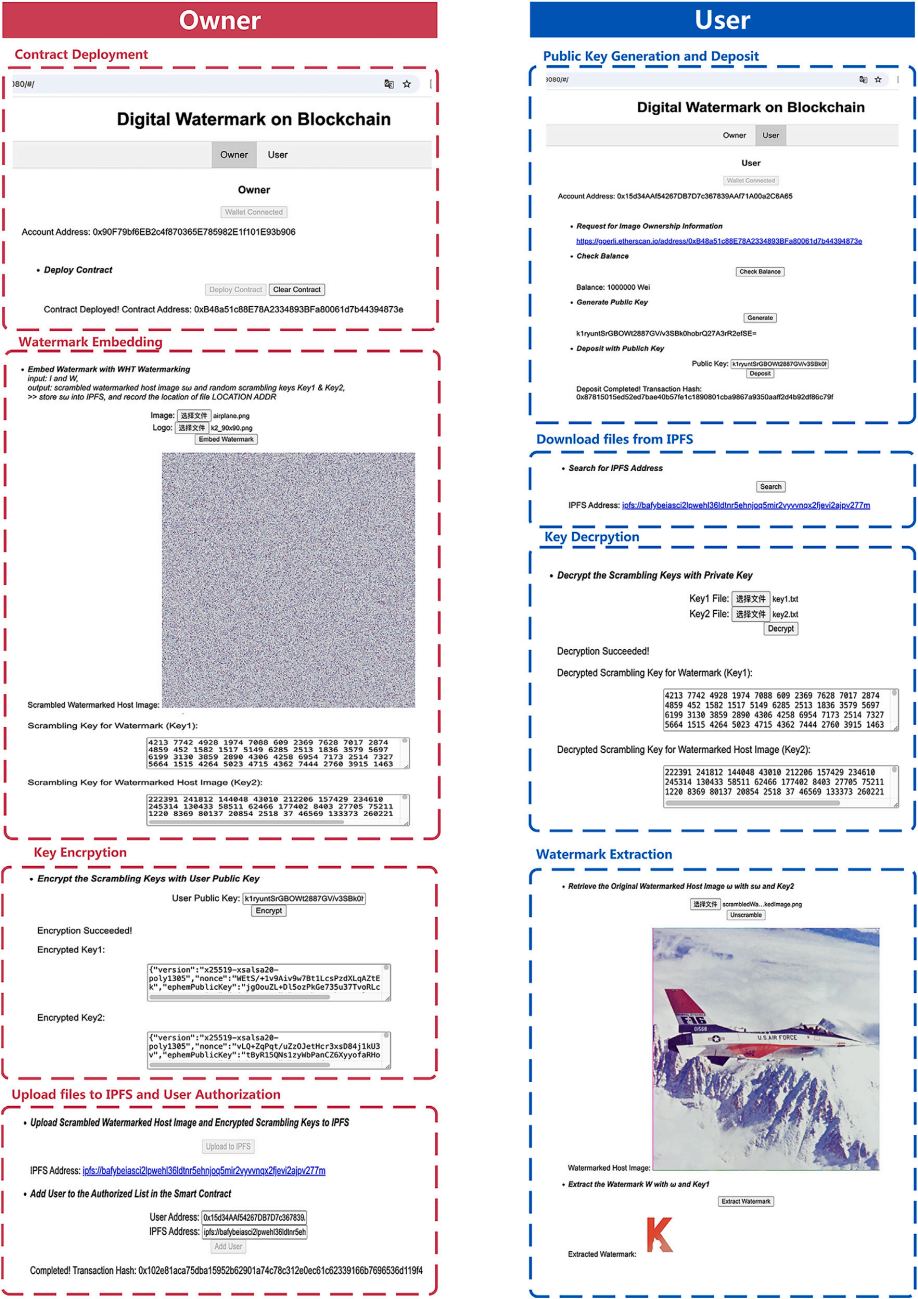
System interface of DApp for the digital watermark on blockchain

To demonstrate the effectiveness of our method, we develop a DApp based on the proposed framework, integrating digital watermarking and blockchain technologies for image copyright protection. The interface of this DApp is illustrated in Figure 7 for explaining its function. Our developed DApp includes two separate interfaces: one for the owner and one for the user. As shown in Figure 7, the owner account has the following four main rights: contract deployment, watermarking embedding, key encryption, as well as upload files to IPFS, and user authorization. On the other hand, the user has the right to generate a public key and deposit, download files from IPFS, decrypt keys, and extract watermarks. First of all, the owner account needs to deploy a new contract for transactions. The address of this contract is “0xB48a51c88E78A2334893BFa80061d7b44394873e.” Users should first generate a public key from their account address using the MetaMask. This public key is then employed to encrypt essential transaction information associated with their wallet address. The generated public key is “k1ryuntSrGBOWt2887GV/v3SBkOhobrQ27A3rR2efSE = ,” which is used for identifying the identity of the verified trader in the following transactions. Users can deposit through contracts deployed by the owner, using the generated public key. The generated transaction hash is “0x87815015ed52ed7bae40b57fe1c1890801cba9867a9350aaff2d4b92df86c79f.” In addition, the user can also check the balance of their balance via smart contract, which is another function for DApp.

In order to protect the copyright of images, the owner should embed the watermark into images by our proposed scheme, thus generating a scrambled watermarked image and two scrambled keys. To protect their security, these keys will be encrypted with the public key, which is obtained from the user’s information via a smart contract. Then, the encrypted keys and the scrambled watermarked image are uploaded to IPFS. To make these files accessible to users, we grant them access to IPFS by adding them to the authorized list. For users, to verify copyright, they download files from the IPFS path, which is accessible when authorized. Using the public key, they can decrypt keys to retrieve the watermarked host image and extract the watermark. The obtained watermarked host image and watermark are shown in Figure 7.

As aforesaid, the proposed method adhered to the concept of decentralization throughout the entire watermarking workflow. The digital image trading and watermark validation are managed by smart contract and the data are transmitted through the Ethereum blockchain, which not only prevents data tampering but also securely controls the authorization to the proper personnel. This is due to the security nature of blockchain. Each block in the blockchain has one unique hash value to verify the blockchain data, if the data were tampered with, the hash value will be accordingly changed. And the hash value is included in the adjacent block, the chain structure makes the data hard to be tampered with. Because any tampering with a block would necessitate recalculating and updating the hashes of all subsequent blocks. Unlike the traditional watermarking method, the proposed method does not rely on third parties for arbitration. This increases both the security and privacy of the data, and also the availability of the framework is more reliable. Moreover, IPFS is used in the proposed solution for data storing and sharing, and asymmetric encryption is applied to secure the stored data. IPFS will store the scrambled watermarked image and the scrambling keys, the keys of which will be encrypted using the public key to the authorized user. In that way, even if unauthorized personnel somehow retrieve the files from IPFS storage, no valuable information would be obtained or leaked without the private key to the authorized user on hand. In summary, the proposed BWM has a high degree of security and availability, which can protect privacy even better than the traditional method.

### Imperceptibility evaluation

**Figure 8:**
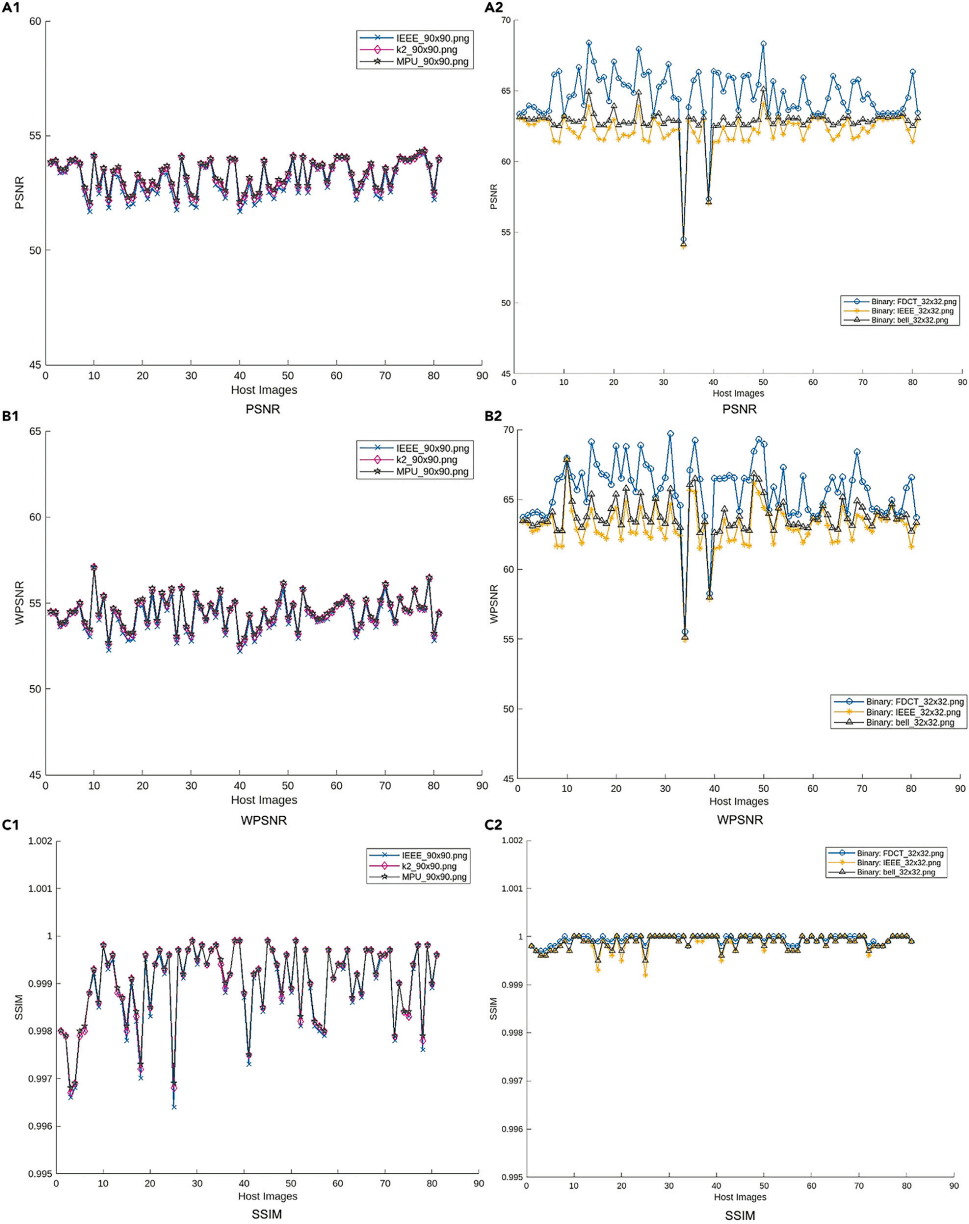
Quality of watermarked images in terms of (A1) (A2) PSNR, (B1) (B2) WPSNR, and (C1) (C2) SSIM $1^{st}$ column: results of watermarked images embedded with color watermark, $2^{nd}$ column: results of watermarked images embedded with a binary watermark.

**Figure 9:**
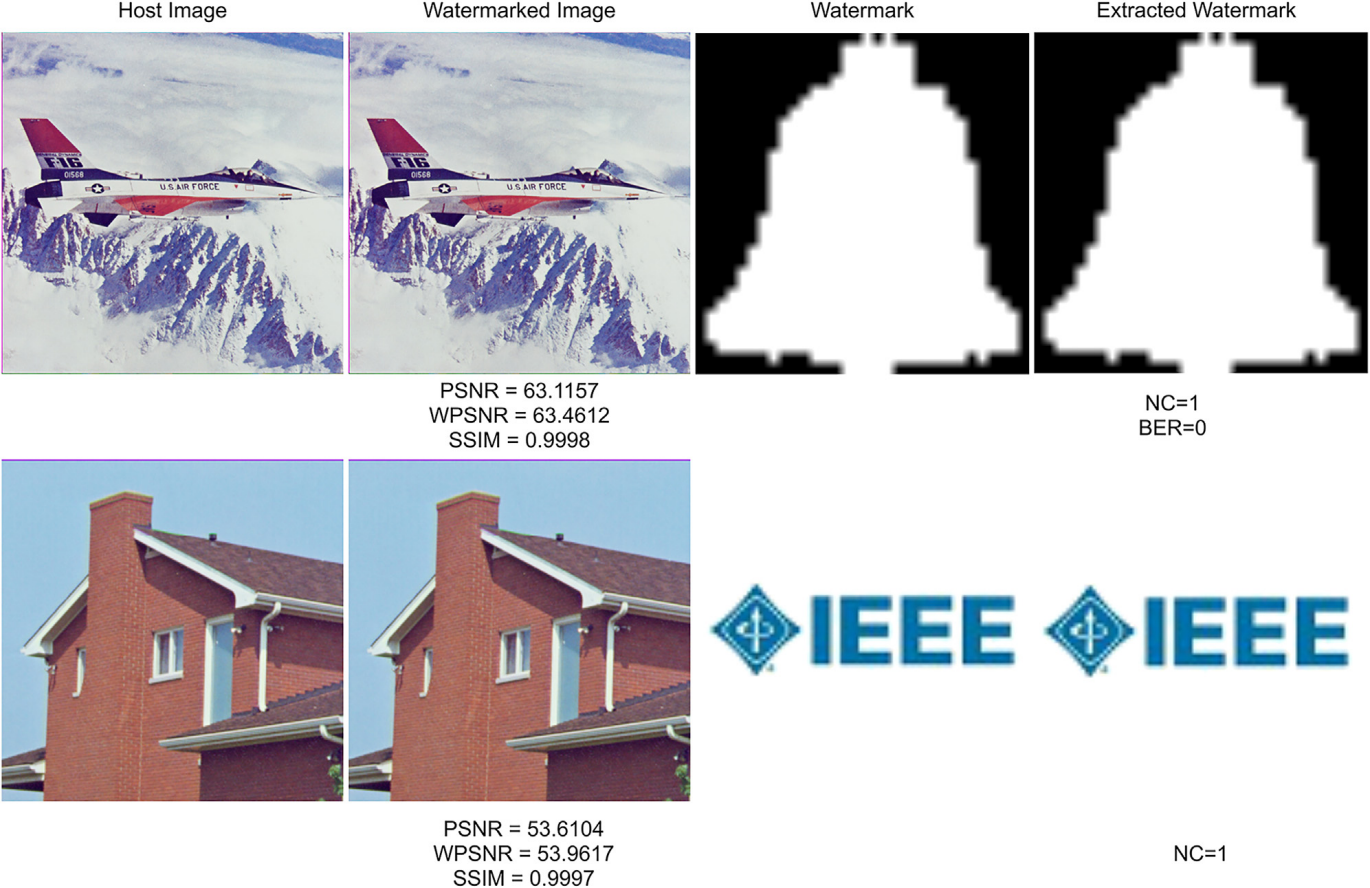
A visualization of watermark embedding and extraction

To make the evaluation more representative and reliable, we use host images from two datasets USC - SIPI Image Database^35^: https://sipi.usc.edu/database/database.php and CVG - UGR Image Database^36^: https://ccia.ugr.es/cvg/dbimagenes/, and they are 24-bit color images of size 512×512. For the watermark images, two categories of images of different sizes, the 24-bit color watermark images in the size of 90 × 90: “MPU,” “IEEE,” and “K2,” and the binary images of size 32×32, “FDCT,” “IEEE,” and “Bell,” are respectively embedded into those host images to test the imperceptibility of the watermarked images. Figure 8 displays the imperceptibility performance of the watermarked image in terms of PSNR, WPSNR, and SSIM respectively. In Figure 8, the $1^{st}$ column shows the quality of watermarked images embedded with color watermark images, where it can be seen that the PSNR values of the watermarked host images are greater than 51.67 dB, with the highest case reaching 54.34 dB; the WPSNR values are all greater than 52.16 dB and the highest exceeds 57.08 dB; and the SSIM values are all higher than 0.9964 and there is one case equal to 0.9999. While the $2^{nd}$ column shows the quality of watermarked images embedded with binary watermark images, where the PSNR values are greater than 53.98 dB and the best results in 68.40 dB, the WPSNR values range from 53.98 to 69.69 dB, and the SSIM values are all greater than 0.9992 and most of them are very close to 1. The test results indicate that the generated watermarked image with the proposed method is imperceptible and has less distortion. The experimental results indicate that fewer watermark payloads achieve better imperceptibility of watermarked images. Moreover, in conjunction with this image quality assessment, the accuracy of watermark extraction could achieve 100% under the ideal condition that there are no attacks. For all the extracted watermarks, the NC is 1, and the BER is 0. For a clearer illustration, two examples are selected for visualization in Figure 9. By comparing with the host image and watermarked image, it is evident our work has excellent imperceptibility. Under this image quality, it can be seen that the extracted watermark is the same as the watermark embedded under the condition without attacks, indicating the effectiveness of watermark extraction. Moreover, to figure out how much of the watermark can be embedded in the image, we test the accuracy of watermark extraction at various capacity rates in Table 1. When the bpp is less than 0.25, the watermark can be accurately extracted. Therefore, the maximum capacity for our watermarking algorithm is 0.25 bpp.

**Table 1:** Watermark extraction accuracy at various embedding capacities (bpp)

Capacities (bpp)	0.1099	0.1495	0.1953	0.2472	0.3052
BER	0	0	0	0	0.5844

### Robustness of watermarking scheme

**Figure 10:**
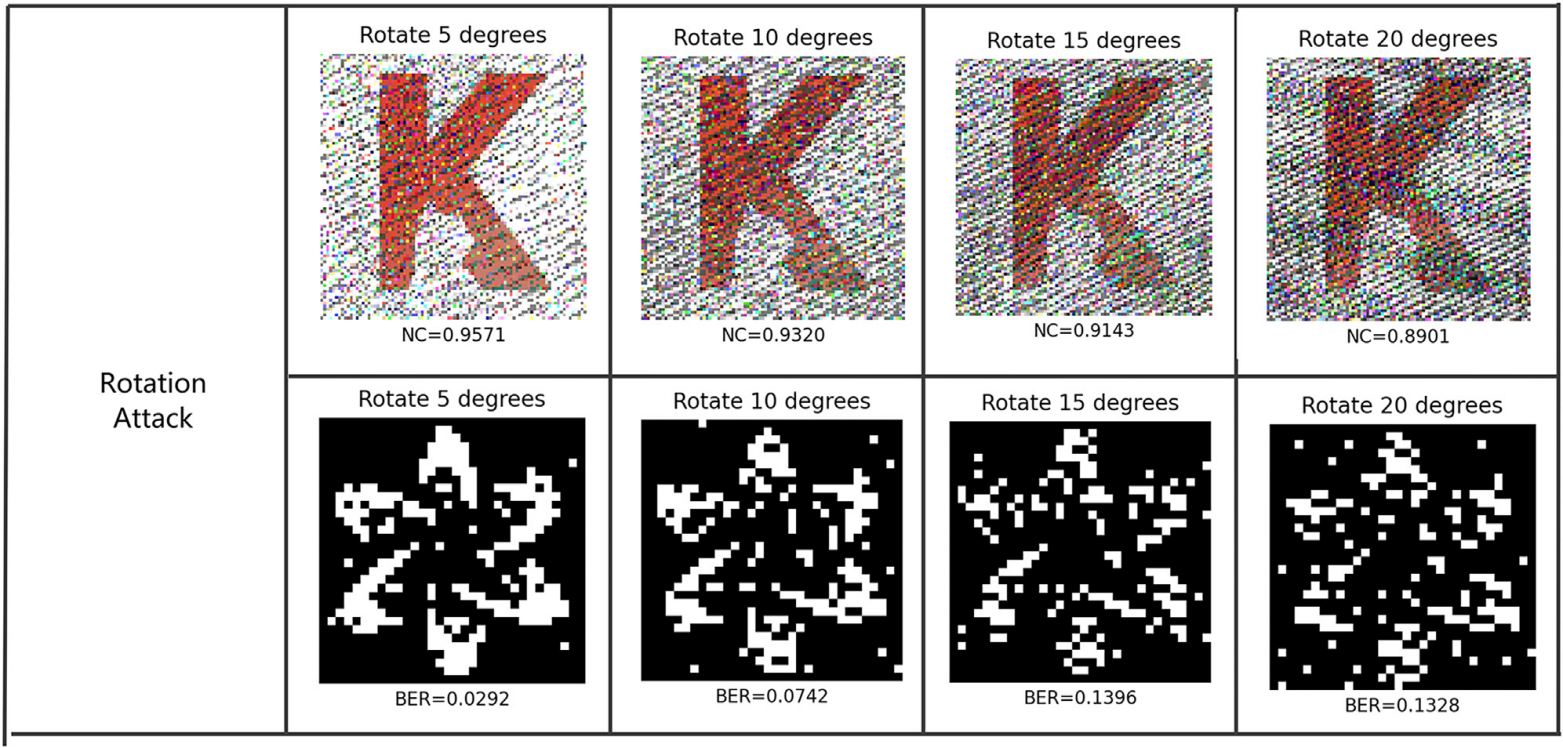
Robustness against rotation attack

**Figure 11:**
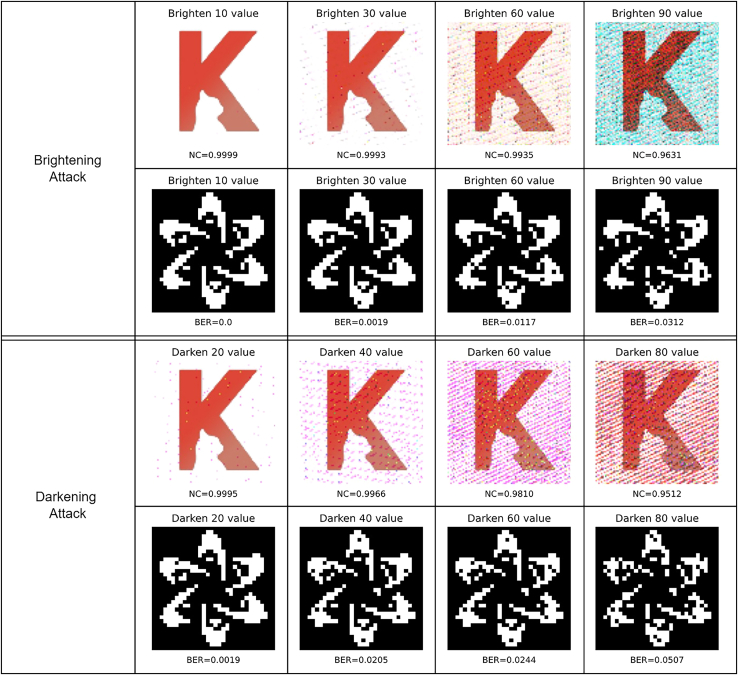
Robustness against intensity change attacks, including Brightening attack and Darkening attack

**Figure 12:**
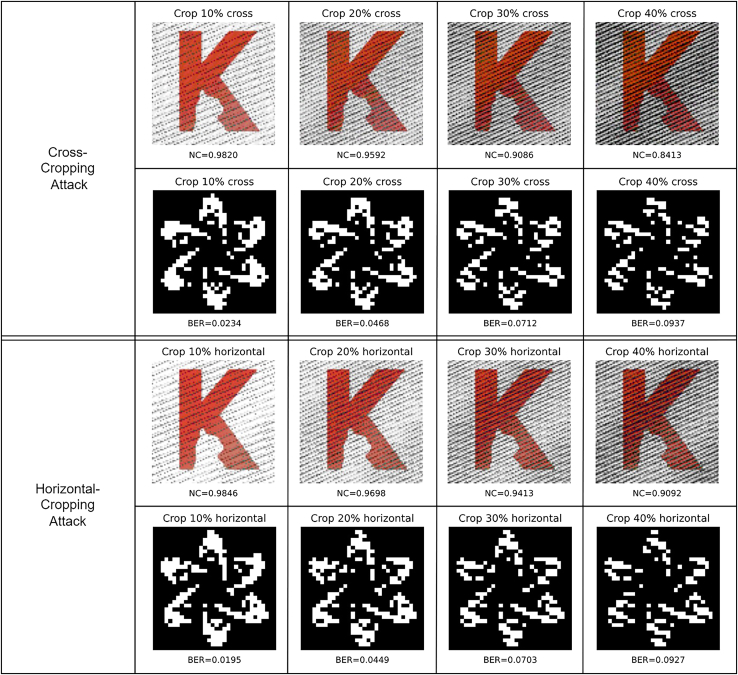
Robustness against cropping attacks, including cross-cropping attacks and horizontal-cropping attack

**Figure 13:**
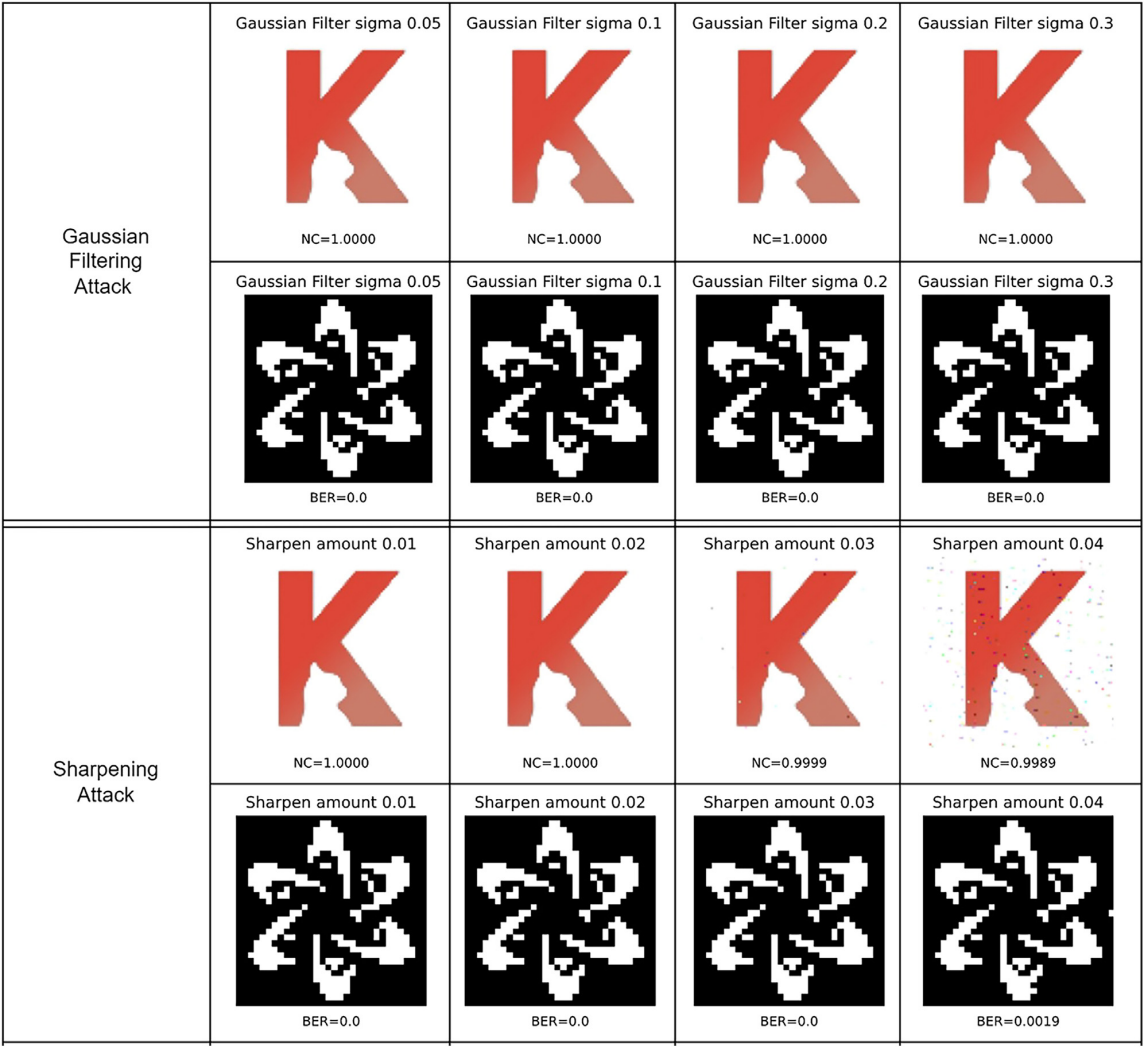
Robustness against Gaussian filtering attack and Sharpening attack

**Figure 14:**
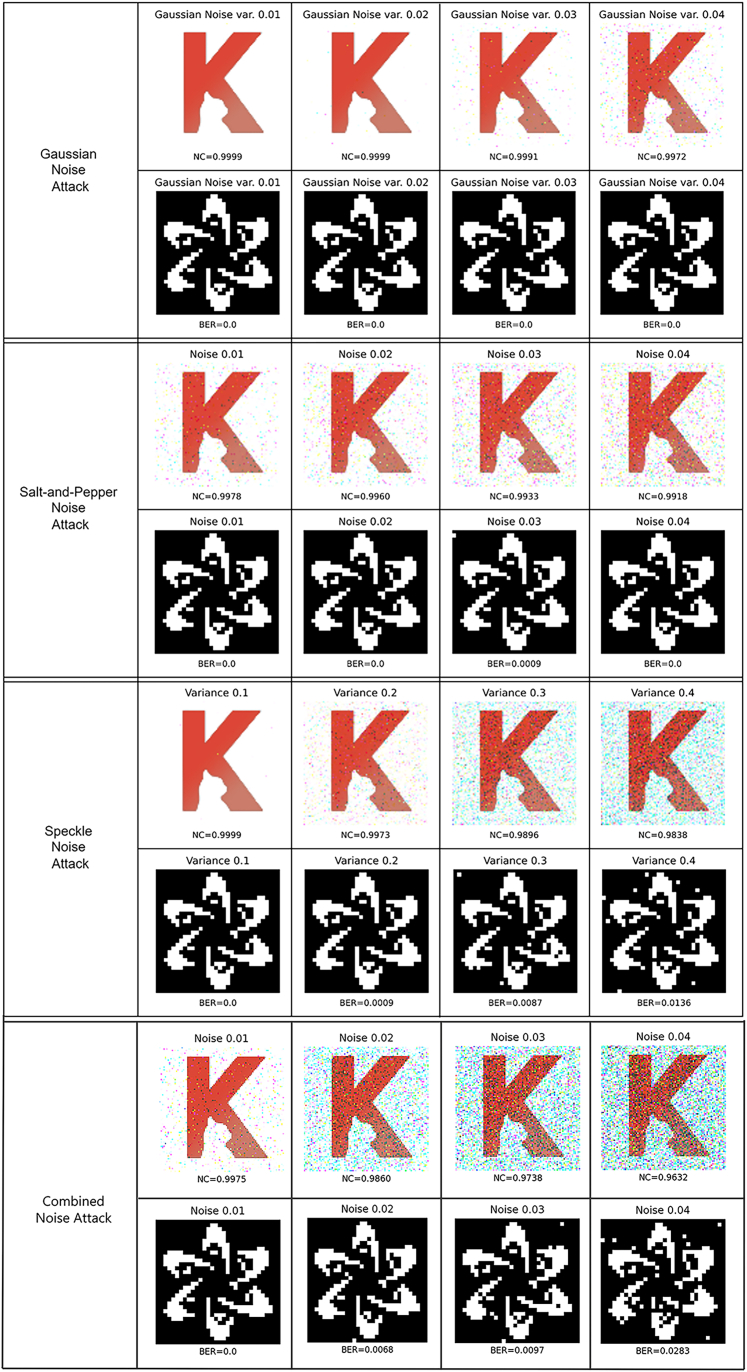
Robustness against noise addition attacks, including Gaussian noise, “Salt-and-Pepper” noise, Speckle noise, and Combined noise

Variant image attacks are carried out to test the robustness of the proposed watermarking method. The color watermark image “K2” and the binary watermark image “FDCT” are respectively embedded into the host images and extracted from the attacked images. Various types of attacks are simulated and employed in these experiments, including rotating the image, changing the image’s intensity, cropping it to remove important details, applying a Gaussian filter to obscure its meaning, and adding noise to reduce the clarity. The corresponding results are shown in Figures 10, 11, 12, 13, and 14, where the NC of the extracted color watermark “K2,” and the BER of the extracted binary watermark “FDCT,” in the various conditions, are calculated accordingly.

Figure 10 shows the robustness against rotation attack. In these tests, angle degrees of 5, 10, 15, and 20 have been applied to rotate the image around its center point, where the corresponding NC values of the extracted “K2” are 0.9801, 0.9573, 0.9429, and 0.9317, and the corresponding BER values of the extracted “FDCT” are 0.0205, 0.0371, 0.0585, and 0.0712. The performance degrades with increased angles of rotation.

Figure 11 shows the robustness against intensity change attacks at different levels, including brightening and darkening attacks. Specifically, 10, 30, 60, and 90 are respectively added to brighten the image, the corresponding NC values of the extracted “K2” are 0.9999, 0.9993, 0.9935, and 0.9631, and the corresponding BER values of the extracted “FDCT” are 0, 0.0019, 0.0117, and 0.0312. For darkening attack, 20, 40, 60, and 80 are respectively deducted from the image, in which the resulting NC values of the extracted “K2” are 0.9995, 0.9966, 0.9810, and 0.9512, and the resulting BER values of the extracted “FDCT” are 0.0019, 0.0205, 0.0244, and 0.0507.

Figure 12 shows the robustness against cropping attacks under different areas, including cross-cropping and horizontal-cropping attacks. In the cross-cropping attacks, 10%, 20%, 30%, and 40% are respectively cropped from the image in both horizontal and vertical directions, where the corresponding NC values of the extracted “K2” are 0.9820, 0.9592, 0.9086, and 0.8413, and the corresponding BER values of the extracted “FDCT” are 0.0234, 0.0468, 0.0712, and 0.0937. The same intensity levels are used in horizontal cropping attacks, which results in better performance than cross-cropping attacks as in line with expectation, where the corresponding NC values of the extracted “K2” are 0.9846, 0.9698, 0.9413, and 0.9092, and the corresponding BER values of the extracted “FDCT” are 0.0195, 0.0449, 0.0703, and 0.0927.

Figure 13 evaluates the robustness against Gaussian filtering attack and sharpening attack. In the Gaussian filtering attacks, Sigmas 0.05, 0.1, 0.2, and 0.3 are respectively applied to filter the image, the NC values of the extracted “K2” are exactly 1 for all results, and the BER values of the extracted “FDCT” are all 0. In the Sharpening attacks, intensities 0.01, 0.02, 0.03, and 0.04 are chosen, where the corresponding NC values of the extracted “K2” are 1, 1, 0.9999, and 0.9989, and the corresponding BER values of the extracted “FDCT” are 0, 0, 0, and 0.0019.

Figure 14 shows the robustness against noise addition attacks, including Gaussian noise, “Salt-and-Pepper” noise, Speckle noise, and Combined noise attacks. The Gaussian noise attacks are applied with variances 0.01, 0.02, 0.03, and 0.04, where the corresponding NC values of the extracted “K2” are 0.9999, 0.9999, 0.9991, and 0.9972, and the BER values of the extracted “FDCT” for all cases are 0. The same intensity levels are used in the Salt-and-Pepper attacks, where the corresponding NC values of the extracted “K2” are 0.9978, 0.9960, 0.9933, and 0.9918, and the corresponding BER values of the extracted “FDCT” are 0, 0, 0.0009 and 0. Next, the Speckle noise attacks are applied with variances 0.1, 0.2, 0.3, and 0.4 respectively, where the corresponding NC values of the extracted “K2” are 0.9999, 0.9973, 0.9896, and 0.9838, and the BER values of the extracted “FDCT” are 0, 0.0009, 0.0087, and 0.0136. Finally, considering that images need to be transmitted over networks, a more complex type of attack, known as a combined noise attack, is applied to the watermarked image. Each type of combined noise attack is overlaid by the following three types: Gaussian noise, “Salt-and-Pepper” noise, and Speckle noise. The corresponding NC values of the extracted “K2” are 0.9975, 0.9860, 0.9738, and 0.9632, and the BER values of the extracted “FDCT” are 0, 0.0058, 0.0097, and 0.0283.

In summary, the experimental results show that the proposed watermarking method is robust against most types of attacks. However, our method shows relatively weaker robustness to rotation attacks, particularly when subjected to large-angle rotations. While our watermarking technique maintains its effectiveness for rotations within a modest range of 10°, its performance degrades with increased angles of rotation.

### Comparison with existing works

**Figure 15:**
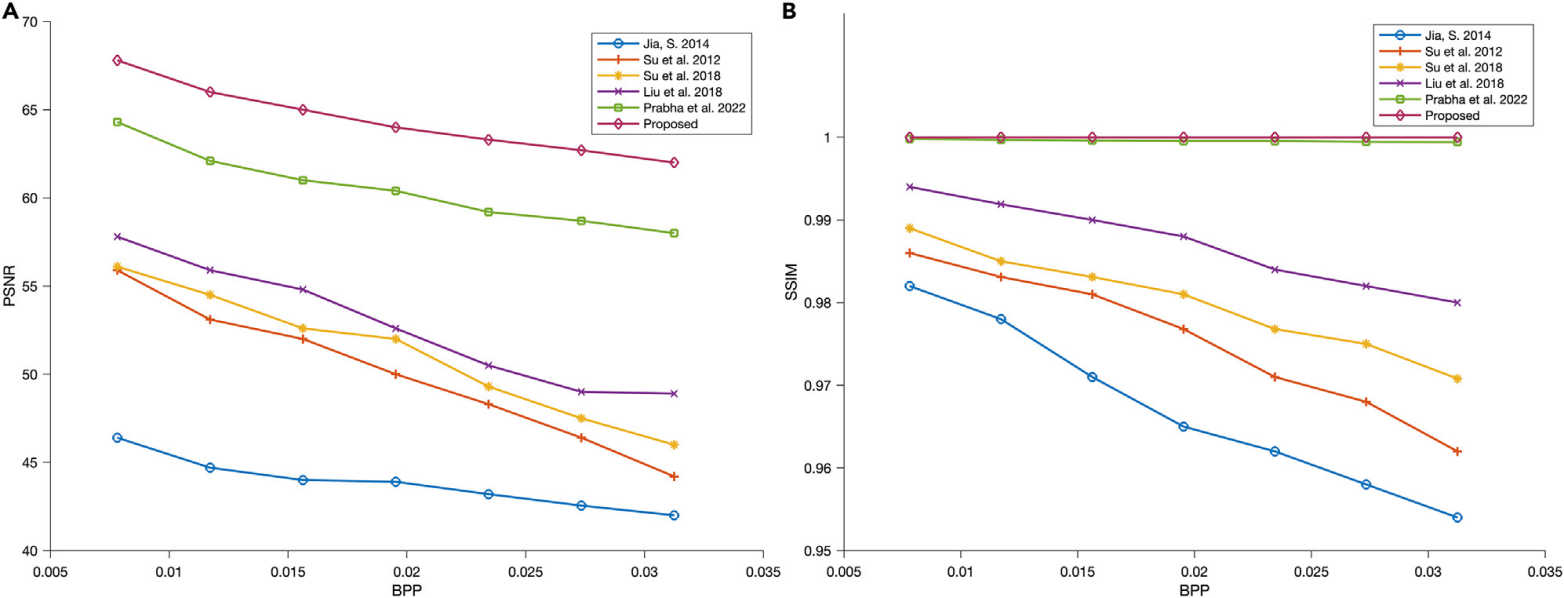
Comparison of capacities and the corresponding quality of watermarked images in terms of (A) PSNR, and (B) SSIM

This section compares the experimental results of the proposed method with the state-of-the-art works. On the aspect of embedding capacities, the proposed scheme is compared with the Jia et al.,^37^ Su et al.,^38^ Su et al.,^39^ Liu et al.,^40^ and Prabha et al..^22^ Experiments are conducted under various capacities, with bpp = 0.03125, 0.02734375, 0.0234375, 0.01953125, 0.015625, 0.01171875 and 0.0078125, and the quality of corresponding watermarked images are evaluated in terms of PSNR and SSIM, respectively, as shown in Figure 15. According to the experimental result, with an increase in bpp, the PSNR and SSIM of watermarked images decreases, but compared to other methods, our proposed method has remarkable performances in both PSNR and SSIM measurements.

In addition to the watermark capacities, we also compare the proposed watermarking method with the existing works on the quality of watermarked images. For a fair comparison, the watermark is embedded into the same cover image. And three images “Airplane,” “Lena,” and “Pepper” are selected as the host images for comparisons, the results are shown in Table 2. In Table 2, with the same set of host images, the PSNR values measured for the watermarking using the proposed scheme have outperformed the watermarking method proposed by Prabha et al.,^22^ Su et al.,^39^ Zhou et al.^41^ and Duan et al..^42^ In addition, in order to comprehensively evaluate the imperceptibility of our work, in Table 3, we calculate the average PSNR and SSIM of watermarked images for comparing with more state-of-the-art deep learning-based approaches.^43^^,^^44^^,^^45^^,^^46^^,^^47^ These metrics are widely recognized for evaluating the quality of watermarked images, with higher values indicating less perceptible changes. The best results are highlighted in bold. The experimental results demonstrate that our work has better imperceptibility than others.

**Table 2:** Comparison on quality of watermarked images in terms of Peak Signal-to-Noise Ratio (PSNR)

	“Airplane”	“Lena”	“Peppers”
Su et al. (2018)^39^	49.9	50	50
Zhou et al. (2018)^41^	43.9	45.5	44.9
Duan et al. (2020)^42^	45.5	45.6	45.8
Prabha et al. (2022)^22^	49.2355	49.2075	49.2645
Proposed Scheme (Color Watermark)	54.0404	54.0940	54.2242
Proposed Scheme (Binary Watermark)	**63.3612**	**63.4841**	**63.3725**

The best results are highlighted in bold.

**Table 3:** Comparison of imperceptibility using average peak signal-to-noise ratio (PSNR) and structural similarity index measure (SSIM) of watermarked images

	PSNR	SSIM
Rahim et al. (2018)^43^	32.9	0.87
Ahmadi et al. (2020)^44^	47.52	0.996
Wei et al. (2020)^45^	37.91	0.979
Zhong et al. (2020)^46^	39.72	–
Ding et al. (2021)^47^	38	0.99
Mahapatra et al. (2023)^48^	31.34	0.994
Proposed Scheme (Color Watermark)	52.8457	0.9985
Proposed Scheme (Binary Watermark)	**64.5425**	**0.9999**

The best results are highlighted in bold.

In Table 4, the NC comparisons between Prabha et al.^22^ and the proposed scheme under variant intensity change attacks are shown. In particular, the proposed scheme has better results in all the comparing attacks, but it is only slightly behind in the dark attack with 20 delta values. Overall, the proposed scheme generates better results, and therefore it can be concluded that the proposed scheme will provide the watermarked images with better robustness compared to the method proposed by Prabha et al..^22^

**Table 4:** Comparison of robustness in terms of quality of extracted watermark image under various attacks

Attacks		Prabha et al. (2022)^22^	Proposed Scheme
Rotation	10	0.9378	**0.9573**
	20	0.892	**0.9317**
	30	0.8662	**0.9141**
	40	0.856	**0.8925**
“Salt-and-pepper” Noise Addition	0.01	0.9971	**0.9976**
	0.02	0.9938	**0.9957**
	0.03	0.9908	**0.9935**
	0.04	0.987	**0.9915**
Median filtering	3×1	0.837	**0.9063**
	5×1	0.725	**0.8025**
	7×1	0.6909	**0.7738**
	9×1	0.6719	**0.7371**
Cropping	25%	0.8661	**0.9551**
	40%	0.7813	**0.9092**
	50%	0.7068	**0.8692**
	60%	0.6385	**0.8239**
JPEG2000	02:01	0.8577	**0.8586**
	03:01	0.8577	**0.8586**
	04:01	0.8577	**0.8586**
	05:01	0.8577	**0.8586**
Brighten	Adding_10	0.9999	**0.9999**
	Adding_30	0.9936	**0.9993**
	Adding_60	0.8918	**0.9935**
	Adding_90	0.8282	**0.9631**
Darken	Reducing_20	**0.9997**	0.9995
	Reducing_40	0.9951	**0.9966**
	Reducing_60	0.9775	**0.981**
	Reducing_80	0.9372	**0.9512**

The best results are highlighted in bold

## Conclusions and future work

In this article, a novel digital watermarking method is presented which is based on Ethereum blockchain, Smart Contract, and IPFS, with an enhanced blind color FWHT algorithm used for watermark embedding and extraction. The proposed method aims to address the limitations of conventional digital watermarking methods by leveraging the immutability and decentralization features of blockchain technology. The Smart Contract and IPFS are used to manage and store the watermark data, respectively, while the enhanced blind color FWHT algorithm is used to embed the watermark into the host image without affecting its visual quality. Experiments are conducted to evaluate the performance of the proposed method and compare it with other watermarking methods. The results show that the proposed method outperforms most of the existing methods in terms of imperceptibility, robustness, and security. However, the proposed method is behind when compared to some other watermarking approaches while processing with a binary watermark. In conclusion, the proposed method offers a promising solution to the challenges faced by existing digital watermarking methods. It provides a secure and privacy-preserving way to protect the ownership and authenticity of digital content, which is becoming increasingly important in the digital age. It is believed that the proposed digital watermarking method has great potential for practical applications and can contribute to the development of the field of digital watermarking. Further optimizations in the aforesaid areas will make the proposed method even more reliable and extensible in actual applications.

### Limitations of the study

Our method exhibits excellent robustness against a series of attacks, which shows its practicality in real-world scenarios. However, it is relatively weaker to resist rotation attacks, particularly when subjected to large-angle rotations. Although our watermarking technique maintains its effectiveness for rotations within a range of 10°, the performance degrades with increased rotation angles.

## Resource availability

### Lead contact

Further information for resources and materials should be directed to and will be fulfilled by the lead contact, Dr. Xiaochen Yuan (xcyuan@mpu.edu.mo).

### Materials availability

This study did not generate new unique reagents.

### Data and code availability

• All experimental data is clearly explained in this article.
• This article does not report original code.
• Any additional information required to reanalyze the data reported in this article is available from the lead contact upon request.

## Acknowledgments

This work was supported by the Science and Technology Development Fund of Macau SAR (Grant number 0045/2022/A), and the Macao Polytechnic University (Project No. RP/FCA-12/2022).

## Author contributions

Conceptualization, Tong Liu, Si-Nga Lai, and Xiaochen Yuan; methodology, Tong Liu, Si-Nga Lai, and Xiaochen Yuan; investigation, Tong Liu and Si-Nga Lai; writing – original draft, Tong Liu, and Si-Nga Lai; writing – review and editing, Xiaochen Yuan, Yue Liu, and Chan-Tong Lam; funding acquisition, Xiaochen Yuan; resources, Xiaochen Yuan, Yue Liu, and Chan-Tong Lam; and supervision, Xiaochen Yuan.

## Declaration of interests

The authors declare no competing interests.

## STAR★Methods

### Key resources table

**Table:** 

REAGENT or RESOURCE	SOURCE	IDENTIFIER
**Software and algorithms**		

Matlab R2022b	MathWorks Inc	https://ww2.mathworks.cn/products/matlab.html; RRID：SCR_001622
InterPlanetary File System	Protocol Labs	https://ipfs.tech/
Metamask	ConsenSys Software Inc	https://metamask.io/
The USC-SIPI Image Database	University of Southern California	https://sipi.usc.edu/database/database.php
CVG - UGR Image Database	University of Granada	https://ccia.ugr.es/cvg/dbimagenes/

### Method details

In this study, all the experiments are implemented on a macOS environment with an Apple M2 CPU and 16 GB RAM, using Python 3.10.9 and MATLAB R2022b. We use USC-SIPI Image Database and CVG-UGR Image Database to test the performance of our proposed scheme. All the software and data involved can be publicly available in the key resources table.

### Quantification and statistical analysis

#### Image quality analysis

In order to objectively evaluate the quality of images, we use three different metrics *PSNR*, *WPSNR*, and *SSIM*. The equations for calculating *PSNR*, *WPSNR*, and *SSIM* are as follows:

$$PSNR=10\times \log_{10}\left(\frac{\max\;{\left(I\left(x,y,z\right)\right)}^{2}}{MSE}\right)$$

where MSE represents the mean square error between I(x,y,z) and W(x,y,z), which is:

$$MSE=\frac{1}{3\times M\times N}\sum_{z=1}^{3}\sum_{x=1}^{M}\sum_{y=1}^{N}{\left[I\left(x,y,z\right)-W\left(x,y,z\right)\right]}^{2}$$

$$WPSNR=10\times \log_{10}\left(\frac{\max\;{\left(I\left(x,y,z\right)\right)}^{2}}{WMSE}\right)$$

$$WMSE=\frac{1}{3\times M\times N}\sum_{z=1}^{3}\sum_{x=1}^{M}\sum_{y=1}^{N}NVF\times {\left(I\left(x,y,z\right)-W\left(x,y,z\right)\right)}^{2}$$

where NVF is a normalized function, which is calculated as follows:

$$NVF=\frac{1}{1+\theta \sigma_{I\left(x,y,z\right)}^{2}}$$

SSIM=L(W,I)×C(W,I)×S(W,I)

where L(W,I) is the luminance comparison, C(W,I) is the contrast comparison, and S(W,I) is a saturation comparison.

The imperceptibility performance of the watermarked image in terms of PSNR, WPSNR, and SSIM respectively is given in Figure 8.

#### Robustness analysis

To evaluate the robustness of our scheme under different attacks, we use *NC* and *BER*. The equations for calculating *NC* and *BER* are as follows:

$$NC=\frac{\sum_{z=1}^{3}\sum_{x=1}^{m}\sum_{y=1}^{n}W\left(x,y,z\right)\times \hat{W}\left(x,y,z\right)}{\sqrt{\sum_{z=1}^{3}\sum_{x=1}^{m}\sum_{y=1}^{n}W{\left(x,y,z\right)}^{2}}\times \sqrt{\sum_{z=1}^{3}\sum_{x=1}^{m}\sum_{y=1}^{n}\hat{W}{\left(x,y,z\right)}^{2}}}$$

$$BER=\frac{1}{P\times Q}\left(\sum_{i=1}^{P}\sum_{j=1}^{Q}{\left[W\left(i,j\right)-W^{*}\left(i,j\right)\right]}^{2}\right)$$

The robustness performance of the proposed method against various attacks are respectively given in Figures 10, 11, 12, 13, and 14.
